# Effect of Bilayer CeO_2−x_/ZnO and ZnO/CeO_2−x_ Heterostructures and Electroforming Polarity on Switching Properties of Non-volatile Memory

**DOI:** 10.1186/s11671-018-2738-4

**Published:** 2018-10-11

**Authors:** Muhammad Ismail, Ijaz Talib, Anwar Manzoor Rana, Tahira Akbar, Shazia Jabeen, Jinju Lee, Sungjun Kim

**Affiliations:** 1 School of Electronics Engineering, Chungbuk National University, Cheongju, 28644 South Korea; 2 Department of Physics, Bahauddin Zakariya University, Multan, 60800 Pakistan

**Keywords:** Heterostructure, Resistive switching, Effect of polarity, Cerium oxide, Schottky emission, Conduction mechanism

## Abstract

Memory devices with bilayer CeO_2−x_/ZnO and ZnO/CeO_2−x_ heterostructures sandwiched between Ti top and Pt bottom electrodes were fabricated by RF-magnetron sputtering at room temperature. N-type semiconductor materials were used in both device heterostructures, but interestingly, change in heterostructure and electroforming polarity caused significant variations in resistive switching (RS) properties. Results have revealed that the electroforming polarity has great influence on both CeO_2−x_/ZnO and ZnO/CeO_2−x_ heterostructure performance such as electroforming voltage, good switching cycle-to-cycle endurance (~ 10^2^), and ON/OFF ratio. A device with CeO_2−x_/ZnO heterostructure reveals good RS performance due to the formation of Schottky barrier at top and bottom interfaces. Dominant conduction mechanism of high resistance state (HRS) was Schottky emission in high field region. Nature of the temperature dependence of low resistance state and HRS confirmed that RS is caused by the formation and rupture of conductive filaments composed of oxygen vacancies.

## Background

Conventional flash memories are facing their physical and practical limits, so searching of new possible candidates for non-volatile memory applications has become very much necessary. Regarding this, several new memory types have been suggested as the next-generation non-volatile memory candidates [1, 2]. Among these, resistive random access memory (RRAM) is being considered as the best candidate for the replacement of conventional memories due to its unique features such as high scaling capability, long memory holding time, smaller device size, fast switching speed, low energy utilization, non-volatility, and simple structure [3]. The memory cell of RRAM is a capacitor-like, metal-oxide-metal (MOM) structure. The bipolar resistive switching (BRS) and unipolar RS (URS) behaviors between two resistance states, i.e., low resistance state (LRS) and high resistance state (HRS) of a resistor film, can be achieved by applying external voltage with appropriate magnitude and polarities [4–6].

The switching performance of a RS device depends on the uniformity of SET-voltage, RESET-voltage, and current levels at LRS and HRS [7]. These switching parameters are influenced by the film dielectrics, electrode materials, and fabrication/operation technique. Numerous models have been proposed so far to explain the dependence of switching characteristics upon these parameters. The switching behavior can be categorized either as bulk-limited or interface-limited [8]. For bulk-limited-type switching, switching parameters are strongly dependent upon permittivity of the dielectric films [9]. However, electrode-limited switching is due to electron correlation at the metal-dielectric interface and the work function of electrode materials [10]. The interface between an anode and dielectric film may also affect the RS parameters of a memory device [10, 11].

Among several oxides, ceria (CeO_2_) has been found to be a promising material for RS memory device applications due to its large dielectric constant (~ 26), lower Gibbs free energy (− 1024 kJ/mol), two oxidation (Ce^+ 4^ to Ce^+ 3^) states, and distribution of vacancies (particularly oxygen vacancies) in a non-stoichiometric pattern [12, 13]. On the other hand, zinc oxide (ZnO), due to its exceptional properties, is extensively being used in various applications. It is noted that ZnO is being utilized as a dielectric owing to its optical transparency, wide band gap, chemical stability, and high resistivity (10^5^ Ω-cm) [14]. Recently, bilayer RS memory structures have been proposed to show superior properties over single layer-based devices in terms of reduction of electroforming and/or SET/RESET voltages, uniformity improvement in switching, long endurance, and self-compliance [15]. Xu et al. [16] investigated the RS behavior of ZrO_2_ and ZnO double-layer stacks illustrating that migration of oxygen vacancies depend upon the height of oxide interfacial barrier. RS behavior observed in the bilayer MnO/CeO_2_ structure was proposed to be due to the oxidation and reduction reaction of CeO_2_ as reported by Hu et al. [17]. Yang et al. [18] revealed good resistive switching characteristics of bilayer CuO/ZnO devices as compared to single-layer ZnO-based devices. Park et al. [19] demonstrated more reliable and reproducible RS operation observed in Pt/TiO_x_/ZnO/Pt memory cells than that noted in Pt/ZnO/Pt memory cells. Hsieh et al. [20] described that Ni/ZnO/HfO_2_/Ni devices exhibited bipolar resistive switching behavior with multilevel characteristics during the RESET process. All such improved RS characteristics motivated deep investigations of bilayer either as ZnO/CeO_2_ or as CeO_2_/ZnO heterostructures, since no study on these stacks and the influence of forming polarity on their RS characteristics and their memory performance has yet been reported.

In this work, we have reported the influence of bilayer heterostructure as well as electroforming polarity on the RS properties of ZnO/CeO_2−x_ and CeO_2−x_/ZnO-based memory devices. Results have shown that the positively electroformed CeO_2−x_/ZnO devices and negatively electroformed ZnO/CeO_2−x_ devices demonstrate lower electroforming voltages and much better cycle-to-cycle switching endurance (~ 10^2^) performance. Temperature dependence of LRS and HRS resistances of these bilayer devices with opposite biasing polarities indicates that the observed RS mechanism can be explained by oxygen vacancies-based conducting channels.

## Methods

Two kinds of Ti/CeO_2_/ZnO/Pt and Ti/ZnO/CeO_2_/Pt heterostructure devices were prepared in this work for comparative study. For fabrication of first Ti/CeO_2_/ZnO/Pt heterostructure device, an active layer of ZnO thin film (~ 10 nm) was deposited on commercial Pt/Ti/SiO_2_/Si (Pt) substrates at room temperature by radio frequency (RF) magnetron sputtering using ZnO (99.99% pure) ceramic target. During deposition, RF power of 75 W and pressure of ~ 10 mTorr under Ar:O_2_ (6:18) mixture (flow rate = 24 sccm) were maintained. Then, CeO_2_ layer (5 nm) was deposited on ZnO/Pt by RF magnetron sputtering under the same conditions to form bilayer CeO_2_/ZnO heterostructure. Finally, Pt/Ti top electrode (TE) was deposited on both of these heterostructures by sequential direct current (DC) magnetron sputtering using metal shadow mask. This technique produced circular devices (memory cells) with a diameter of 150 μm. Here, Pt was used as a protective layer to shield Ti TE from oxidation. In the same way, a second Ti/ZnO/CeO_2_/Pt heterostructure device was also fabricated under the same conditions as maintained for Ti/CeO_2_/ZnO/Pt heterostructures. Both Ti/CeO_2_/ZnO/Pt and Ti/ZnO/CeO_2_/Pt heterostructure memory devices were characterized by Agilent B1500A semiconductor parameter analyzer using a standard two-probe measurement method. The bilayer structure of these devices was characterized using cross-view high-resolution transmission electron microscopy (HRTEM-JEM 2001F).

## Results and Discussion

**Fig. 1:**
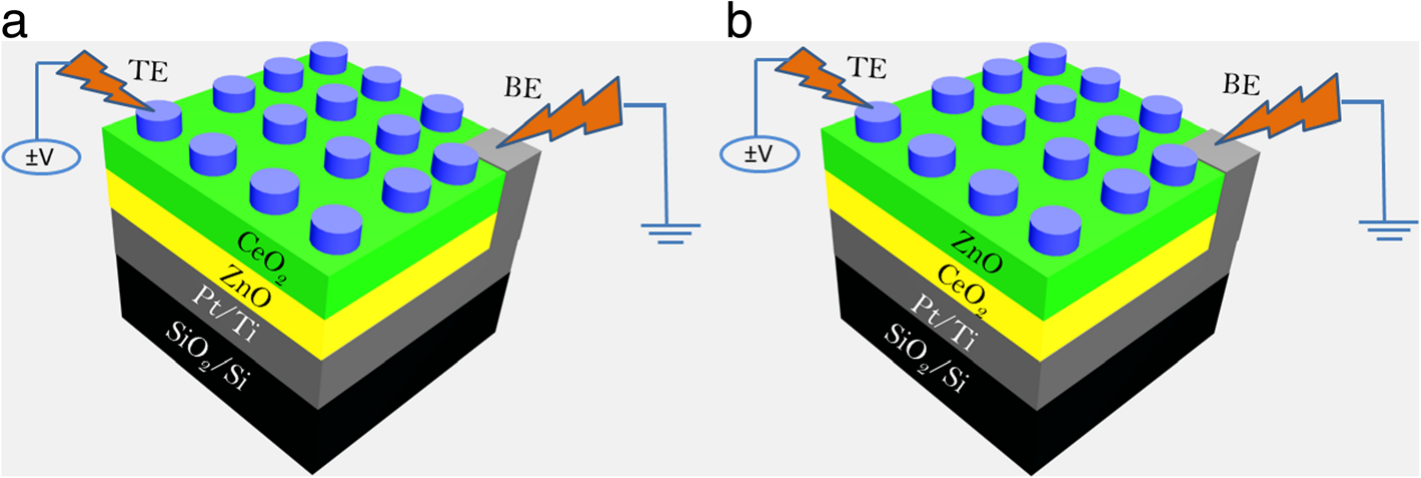
Schematic configuration of the bilayer **a** Ti/CeO_2_/ZnO/Pt and **b** Ti/ZnO/CeO_2_/Pt devices

**Fig. 2:**
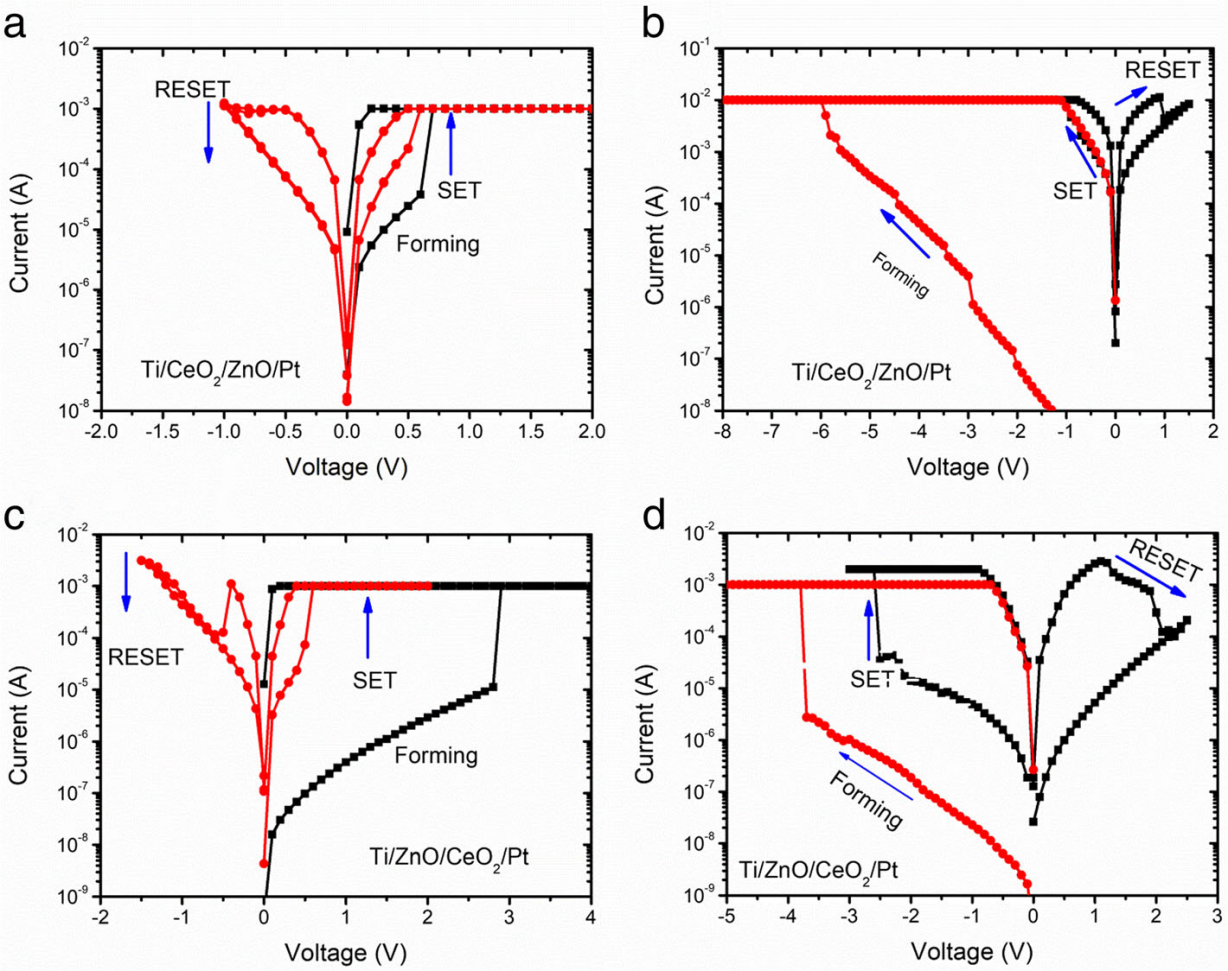
Devices depict the typical bipolar behavior. **a** Positive (+ve) forming and subsequent switching operation and **b** negative (−ve) forming and switching operation of the Ti/CeO_2−x_/ZnO/Pt hetrostructures. **c** +ve forming and switching operation and **d** −ve forming and switching operation of Ti/ZnO/CeO_2−x_/Pt memory devices. Arrows indicate switching directions

Figure 1a, b shows the schematic configuration of bilayer Ti/CeO_2_/ZnO/Pt and Ti/ZnO/CeO_2_/Pt heterostructure memory devices, respectively. Figure 2a–d shows typical current-voltage (*I*-*V*) curves of Ti/CeO_2−x_/ZnO/Pt and Ti/ZnO/CeO_2−x_/Pt heterostructure memory devices, also including the initial electroforming process, indicating typical bipolar RS characteristics. When a + 2 V sweep was applied to TE, sudden jump of current occurred at 0.6 V indicating the formation of conducting paths between two electrodes (Fig. 2a). The device remained in ON-state (LRS) after the positive electroforming voltage was removed. Figure 2a also displays that the device successfully switched back to HRS with a negative voltage sweep from 0 to − 1 V, and to LRS again with a positive voltage sweep from 0 to + 1 V. An opposite polarity, i.e., negative electroforming voltage, was also provided to activate/initiate switching behavior in the same heterostructure memory cell. In this regard, when a 0 to − 8 V sweep was applied to TE, device resistance exhibited a sudden fall at − 5.6 V, thereby turning it ON from OFF-state called negative electroforming (Fig. 2b). After negative electroforming, the device failed to positive RESET and negative SET due to its irreversible breakdown. It is noted that much higher negative electroforming voltages are needed to initiate RS characteristics than positive electroforming voltages. However, after negative electroforming, there was no switching hysteresis observed, as the device stayed in the ON state irrespective of the application of SET and RESET voltages; this fact indicates the formation of permanent conductive filaments during electroforming process. The irreversible breakdown during negative electroforming might be resulted from different tunneling barrier heights initiated by the difference in top and bottom electrode work functions [21]. These results show that the device with Ti/CeO_2−x_/ZnO/Pt heterostructure can be suitable for non-volatile characteristics only if it is electroformed with positive polarity, followed by negative and positive polarities of corresponding RESET and SET operations. The only difference between the second (Ti/ZnO/CeO_2−x_/Pt) and first (Ti/CeO_2−x_/ZnO/Pt) devices is the position of insulating layers in the sandwich heterostructure. That is why the device with Ti/ZnO/CeO_2−x_/Pt heterostructure can also be electroformed at both positive and negative polarities of biasing potentials likewise Ti/CeO_2−x_/ZnO/Pt heterostructure device. Figure 2c shows typical bipolar *I-V* curves for such a positive electroforming and subsequent switching behavior. With 0 to + 4 V sweep, the device was electroformed to switch it to ON state (an abrupt resistance change at + 3 V) as illustrated by Fig. 2c. The device was then switched ON below + 2 V (positive SET) and OFF at − 1.5 V (negative RESET) during repeatable switching cycle. Similarly, the device with the same heterostructure electroformed negatively (at − 3.5 V) showed positive RESET (at + 1.5 V) and negative SET (at − 2.5 V) as obvious from Fig. 2d. To protect both the devices from permanent breakdown, current compliance of 1 mA was applied during electroforming and SET processes.

**Fig. 3:**
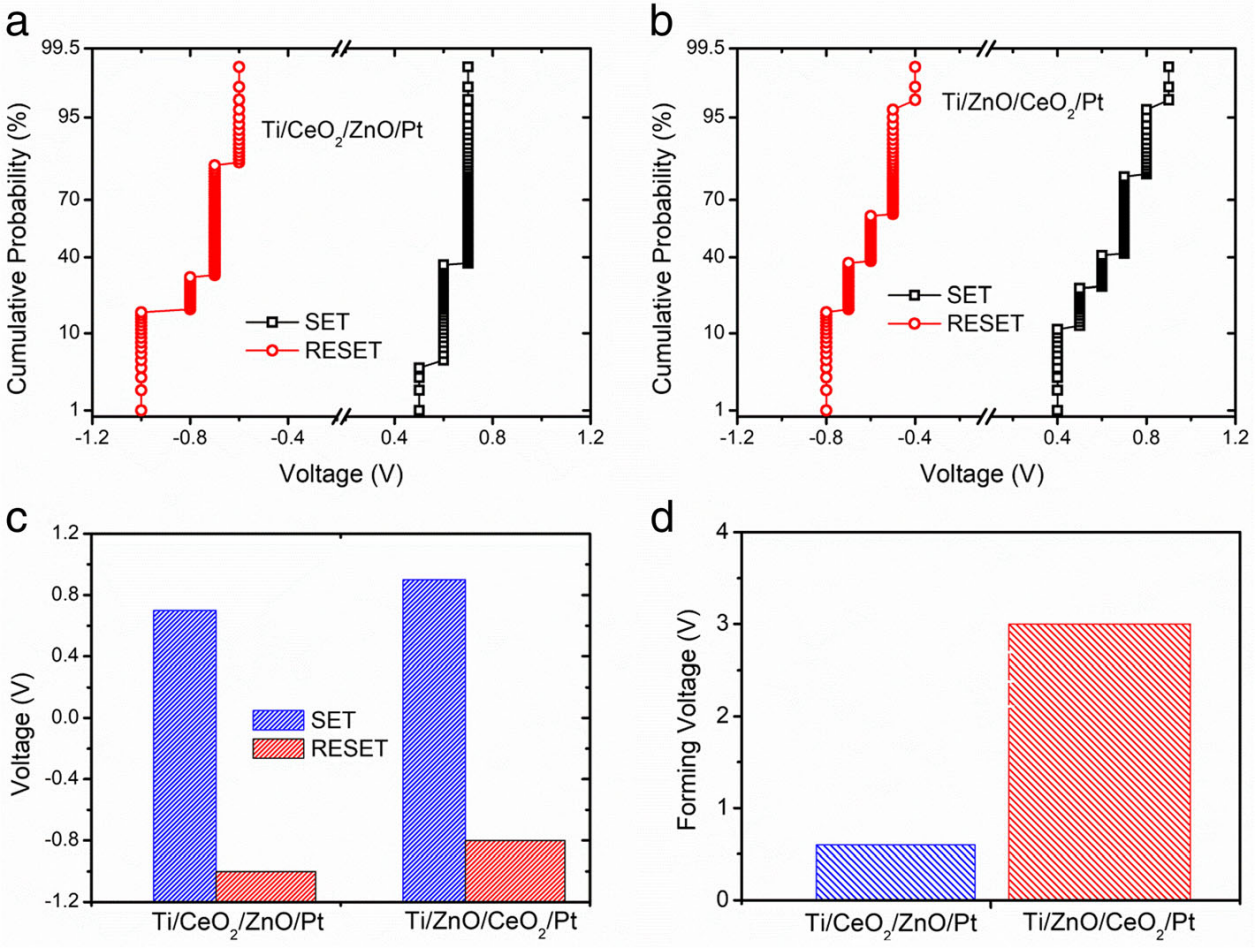
Cycle-to-cycle cumulative probability distribution of operational voltages in **a** Ti/CeO_2−x_/ZnO/Pt and **b** Ti/ZnO/CeO_2−x_/Pt heterostructure memory devices. **c** Statistical analysis of SET and RESET-voltages of Ti/CeO_2−x_/ZnO/Pt and Ti/ZnO/CeO_2−x_/Pt heterostructure memory devices. **d** Statistical evaluation of the electroforming voltages for both Ti/CeO_2−x_/ZnO/Pt and Ti/ZnO/CeO_2−x_/Pt heterostructure memory devices

To check the uniformity of switching parameters for both heterostructure memory devices, cummulative probabilities of operational voltages (SET and RESET voltages) noted in various switching cycles are displayed in Fig. 3a, b. The Ti/CeO_2−x_/ZnO/Pt heterostructure memory device exhibits relatively narrower variations in SET and RESET voltages as compared to Ti/ZnO/CeO_2−x_/Pt heterostructure memory device. Figure 3c, d reveals the statistical analysis of average SET, RESET, and electroforming voltages of both heterostructure memory devices. The Ti/CeO_2−x_/ZnO/Pt devices are found to require much lower electroforming voltages as compared to those needed for Ti/ZnO/CeO_2−x_/Pt heterostructure memory devices, but SET and RESET voltages demonstrate only slightly variations. Smaller fluctuations in operation voltages of both devices might be associated with the creation and rupture of filaments taking place at the interfaces. Liu et al. [22] suggested that the low SET/RESET voltages and switching uniformity noted in WO_x_/NbO_x_ bilayer structure could be attributed to combined effect of oxygen migration between two oxide layers and metal-insulator transition. As Gibbs free energy ΔG of the oxide formation for ZnO and CeO_*x*_ has a huge difference of about 706 kJ/mol (for CeO_2_, ΔG = − 1024 kJ/mol and for ZnO it is − 318.52 kJ/mol) and localized heating effect occurs, the exchange of oxygen is induced. It is well-known that ZnO thin layer has a lot of oxygen vacancies due to low formation energy [23]. Also, many initial oxygen vacancies present in ZnO layer play a major role in conduction via shallow traps [24]. Additionally, it is stated that the forming free phenomenon in ZnO-based devices might be credited to a high concentration of oxygen vacancies already present in ZnO crystals [25]. From all the abovementioned facts, it can be concluded that in the presence of ZnO film possessing a lot of oxygen vacancies in both heterostructure devices (ZnO/CeO_2−x_ and CeO_2−x_/ZnO) plays a crucial character in the reduction of operational voltages. Oxygen vacancies in ZnO might act as shallow traps for electrons and electrons in these trapping sites can easily be trapped or de-trapped at small values of SET and RESET voltages.

**Fig. 4:**
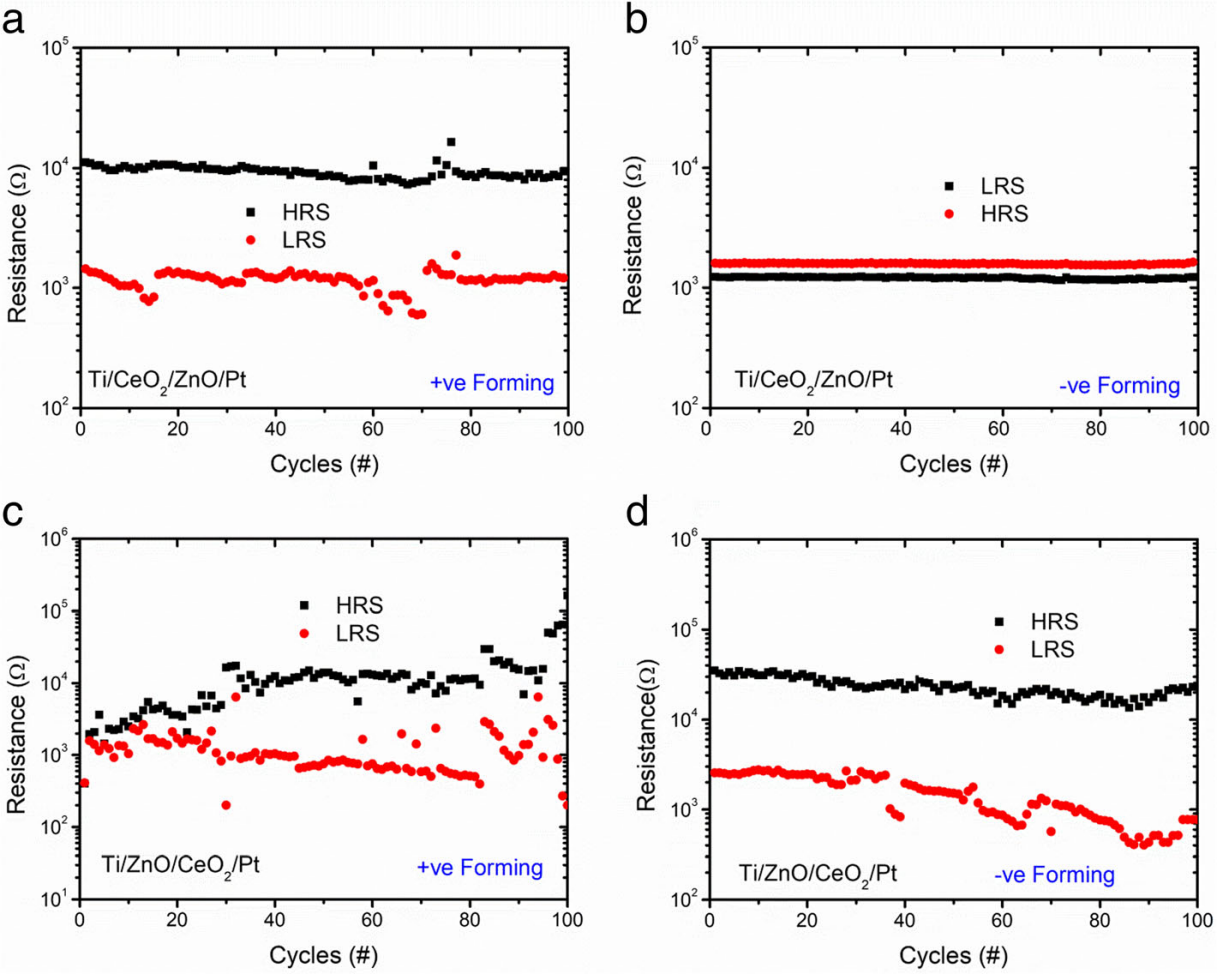
Endurance characteristics of **a** positively electroformed and **b** negatively electroformed Ti/CeO_2−x_/ZnO/Pt heterostructure memory devices. **c** Positively electroformed and **d** negatively electroformed Ti/ZnO/CeO_2−x_/Pt heterostructure memory devices

To investigate the reliability of both device heterostructures, endurance tests at different polarities of biasing potential were performed. The resistance values of HRS and LRS are obtained at 0.2 V from DC endurance switching cycles. Figure 4a describes the endurance characteristics of Ti/CeO_2−x_/ZnO/Pt heterostructure memory device. It is seen that positively electroformed Ti/CeO_2_/ZnO/Pt heterostructure memory devices exhibited good endurance with memory window of ~ 10 that could ensure clearly distinguishable HRS and LRS. Formation of Schottky barrier at Ti/CeO_2−x_ interface is due to work function difference between the Ti TE and the adjacent layer of CeO_2−x_, leading to good RS properties. When the same heterostructure device (Ti/CeO_2−x_/ZnO/Pt) was electroformed negatively, the device could not be changed from LRS to HRS as shown in Fig. 4b. Figure 4c illustrates the endurance characteristics of positively electroformed Ti/ZnO/CeO_2−x_/Pt heterostructure memory device exhibiting very poor endurance property. Memory window appears to be almost collapsed making the ON and OFF states practically indistinguishable. This fact may be attributed to the incapability of ZnO to capture the injected carriers because of the presence of high concentration of vacancies, which makes the conduction track toward Ti TE because no barrier is formed at Ti/ZnO interface due to negligible work function difference between Ti (4.33 eV) and ZnO (4.35 eV), and this leads to poor endurance [26]. Another reason may be the high density of defects within the ZnO/CeO_2−x_ matrix created under strong electric field, because oxygen vacancy migration is significantly enhanced along the extended defects. In addition, positively charged oxygen vacancies segregated at defect sites increase the surface density states, resulting in collapse of ON/OFF ratio. It suggests that when Ti/ZnO blocking contact is formed, Fermi levels are in alignment with each other due to the movement of electrons from Ti to ZnO. As a result, majority carriers are gathered at the surface of oxide layer and almost no barrier is formed [26]. Figure 4d demonstrates much better endurance characteristics of the negatively formed Ti/ZnO/CeO_2−x_/Pt heterostructure memory device as compared to those of positively formed device. Zhu et al. [27] fabricated three different kinds of devices: (i) Ag/ZnO/NSTO/In, (ii) Ag/CeO_2_/NSTO/In, and (iii) Ag/CeO_2_/ZnO/NSTO/In. The bilayer device (CeO_2−x_/ZnO), as compared to single-layer ones, exhibited better RS behavior with data retention of about 10 years. They attributed better RS characteristics of bilayer heterostructures to the interface barrier between CeO_2−x_/ZnO bilayer structure and the existence of large number of vacancies acting as trap centers in ZnO films.

**Fig. 5:**
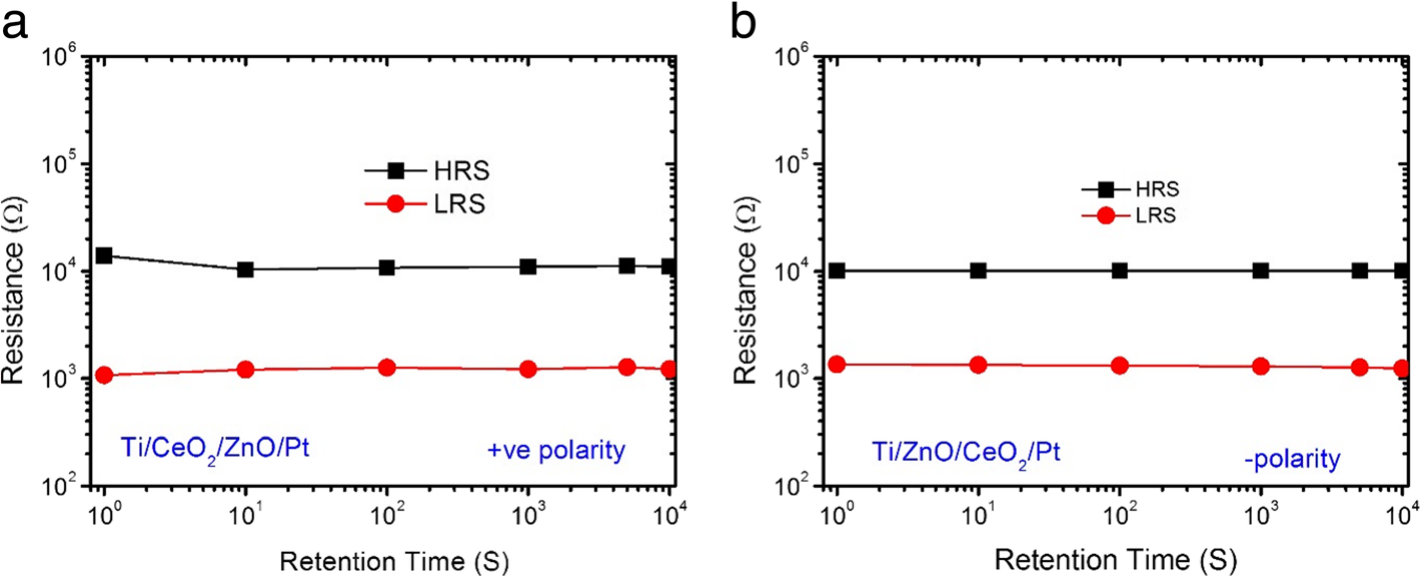
Retention characteristics of **a** positively electroformed Ti/CeO_2−x_/ZnO/Pt heterostructure memory devices and **b** negatively electroformed Ti/ZnO/CeO_2−x_/Pt heterostructure memory devices at room temperature

The retention performance of both CeO_2−x_/ZnO and ZnO/CeO_2−x_ bilayer heterostructures was also investigated. The retention time of both heterostructure devices was measured at room temperature with a reading voltage of 0.2 V as obvious from Fig. 5a, b. No electrical power was needed to maintain resistance constant at any given state. Up to retention time of 10^4^ s, resistances of the HRS and LRS reveal no signs of deterioration at all, implying that the information stored in both heterostructure devices can be kept for much longer times than 10^4^ s.

**Fig. 6:**
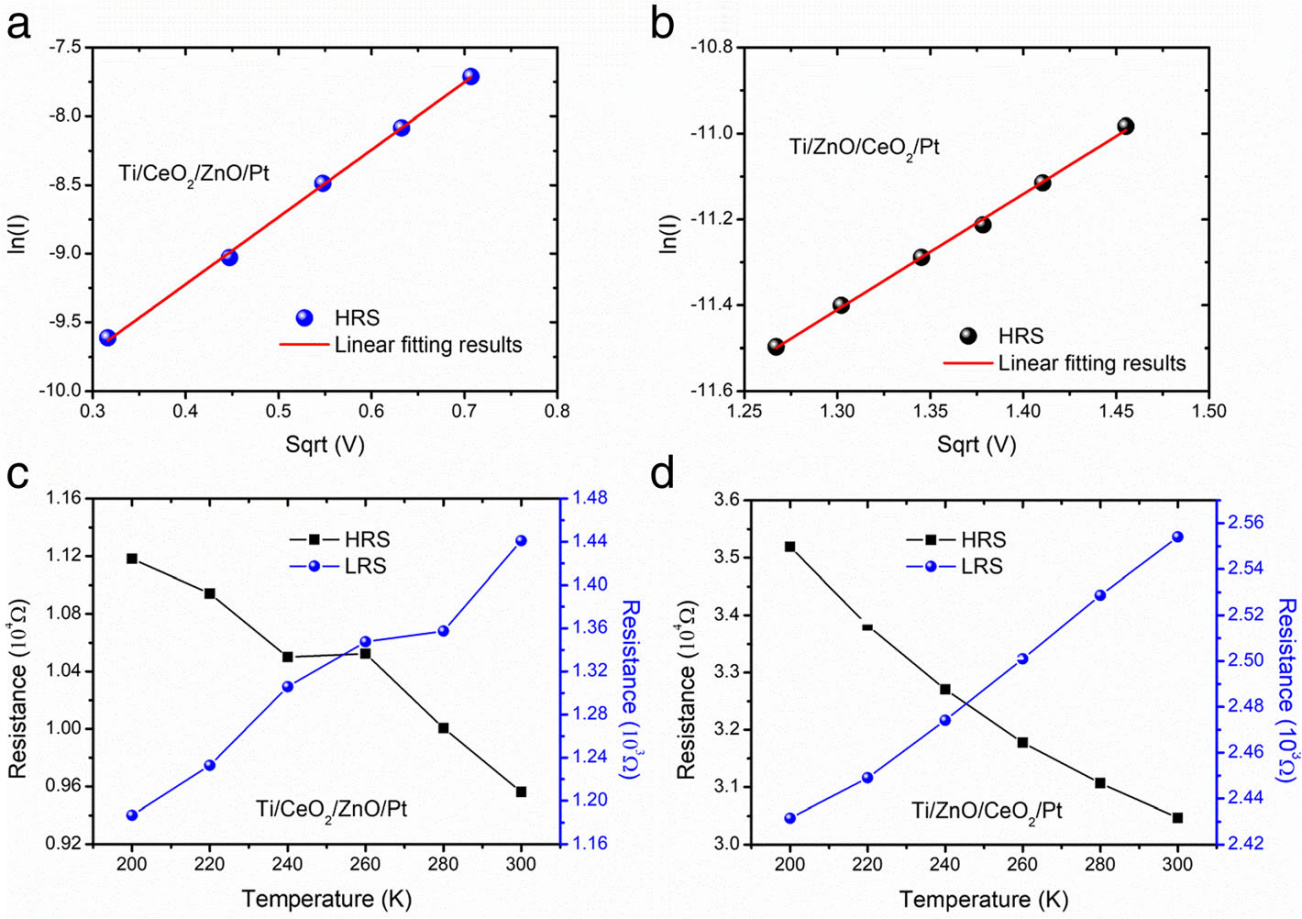
log*I-V*^1/2^ characteristics in the HRS of SET-state. **a** For Ti/CeO_2−x_/ZnO/Pt. **b** For Ti/ZnO/CeO_2−x_/Pt heterostructure memory devices. Temperature dependence of LRS and HRS of **c** Ti/CeO_2−x_/ZnO/Pt and **d** Ti//ZnO/CeO_2−x_/Pt heterostructure memory devices

To investigate about the conduction mechanism prevailing in the high field region of both heterostructure memory devices, curve fitting procedure was performed under positive (for CeO_2−x_/ZnO) and negative (for ZnO/CeO_2−x_) polarities of biasing potential. Figure 6a, b describes that linear curve fittings to experimental data are well aligned with Schottky emission behavior for both heterostructure devices in their respective biasing polarities. Schottky emission is known to take place when electrode injects thermally activated electrons across the barrier into the conduction band of insulator, so it is called electrode-limited mechanism. Generally, Schottky emission arises when electrode contact is highly carrier injective. The linear relation of ln(*I*) vs. √*V* indicates that the electrons have achieved an adequate amount of energy to conquer the energy barrier. Ohmic conduction (current being proportional to applied voltage) occurring at a low field region shows that the current flows due to thermally generated electrons (results are not shown here). The Schottky emission model can usually be described by an equation of the form [28]: $\ln (J)=\ln {A}^{\ast }{T}^2-q\left({\varPhi}_b-\sqrt{\frac{qV}{4\pi {\varepsilon}_o{\varepsilon}_rd}}\ \right)/{k}_BT$, where *J* is current density, A* is Richardson constant, *T* is temperature, *q* is electric charge, *V* is eclectic voltage, *ε*_*r*_ is dielectric constant, *ε*_*o*_ is permittivity of free space, *d* is film thickness, and *k*_*B*_ is Boltzmann constant. Furthermore, temperature-dependent resistance values of LRS and HRS were measured at voltage of 0.2 V in the temperature range of 200–300 K for both CeO_2−x_/ZnO and ZnO/CeO_2−x_ heterostructure memory devices as shown in Fig. 6c, d. It can be noticed that the electrical transport properties of both heterostructure devices in low resistance state are metallic in nature, i.e., resistances in LRS increase with increasing temperature. In contrast to this, electrical transport properties for both the devices at HRS are semiconducting in nature, i.e., resistances in HRS decrease with rising temperatures. Values of activation energy (*E*_a_) obtained from Arrhenius plots of LRS of both heterostructure devices (results not shown) are ~ 0.092 eV, and comparable to energy of the first ionization of oxygen vacancies (~ 0.1 eV) [25, 26, 29], which indicates that the first ionization of oxygen vacancies is responsible for the conduction at HRS, further confirming the dominance of Schottky emission as operative conduction mechanism in the HRS. The metallic behavior in LRS and semiconducting behavior in HRS of both heterostructure devices provide sufficient evidence in the support of switching behavior in Ti/CeO_2−x_/ZnO/Pt and Ti//ZnO/CeO_2−x_/Pt heterostructure memory devices that it can be associated with oxygen vacancies-based conductive filamentary mechanism.

**Fig. 7:**
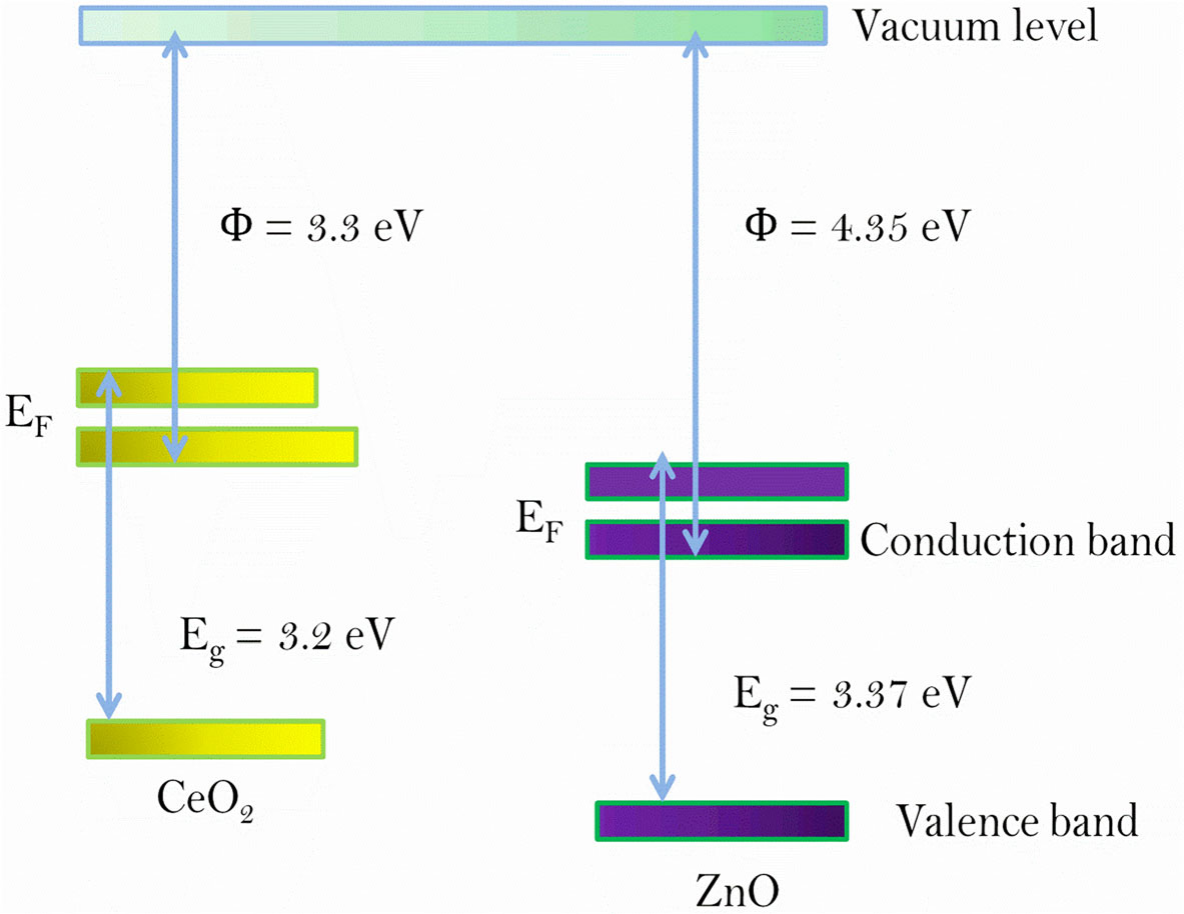
Energy band diagram of CeO_2_ and ZnO n-type semiconducting materials

Figure 7 describes the proposed energy band diagram of CeO_2_ and ZnO n-n-type semiconducting materials in the steady state. The difference between work functions of ZnO (4.35 eV) and CeO_2_ (3.33 eV) is equal to 1.02 eV for the same electronic transition on the oxygen vacancy [30]. The lower work function of CeO_2_ (3.33 eV) than that of ZnO (4.35 eV) enables the movement of electrons from CeO_2_ to ZnO, giving rise to their higher concentration in the matrix.

According to our previous study [31], RS characteristics of single-layer Ti/CeO_2−x_/Pt device were attributed to the formation of a TiO interfacial layer that plays a key character in the creation and rupture of conductive filamentary paths. Warule et al. proposed that RS behavior in the Ti/ZnO/Pt devices was induced by the creation and disconnection of oxygen vacancies-based conductive filaments [32]. In addition, forming-free phenomenon in Ti/ZnO/Pt devices is related with the existence of a considerable amount of oxygen vacancies in the as-prepared Ti/ZnO/Pt devices [32–34]. Schottky barrier at the ZnO/Pt interface can be eliminated by the existence of an adequate amount of oxygen vacancies in the ZnO film, resulting in an Ohmic contact at ZnO/Pt interface. Accordingly, the formation of TiO interfacial layer can be associated with the RS effect in bilayer ZnO/CeO_2−x_ and CeO_2−x_/ZnO heterostructures. It is well known that Ti is highly reactive metal with atmospheric oxygen: therefore, it can easily form TiO layer at Ti/oxide interface [35]. In Ti/ZnO/CeO_2−x_/Pt heterostructure memory device, ZnO is n-type semiconductor and contains a lot of oxygen vacancies in it, so an Ohmic contact is formed at Ti/ZnO interface [36]. As Ti and ZnO have approximately the same work functions, so, Ti is unable to extract oxygen ions from ZnO to create a TiO interfacial layer. It has been reported that non-lattice oxygen ions and oxygens related with lattice defects exist in ZnO films [37]. Due to deposition of ceria (CeO_2_) by RF sputtering at room temperature, fabricated CeO_2_ films are polycrystalline in nature. So ceria films can be non-stoichiometric as we have already proved in our earlier research work that ceria is reduced to CeO_2−x_ [12]. Hu et al. [17] also reported such reduction of CeO_2_ during deposition to CeO_2−x_. Defects in the CeO_2−x_ films are insufficient to mobilize oxygen ions. Therefore, CeO_2−x_ layer serves as oxygen reservoir in Ti/ZnO/CeO_2−x_/Pt heterostructure. Gibb’s energy for formation of CeO_2_ is much smaller (− 1024 kJ/mol) than that of ZnO (− 318.52 kJ/mol) as described earlier, so there exists non-lattice oxygens in ZnO due to its non-stoichiometric nature, which move toward CeO_2_ layer even in the absence of external bias [37]. Therefore, when Ti TE is deposited on ZnO, no interfacial layer is expected to form between Ti and ZnO, although Gibbs energy of formation of TiO is smaller than that of ZnO. When positive voltage is applied to the TE, oxygen ions are attracted toward the CeO_2−x_/Pt interface and conductive filaments are generated with oxygen vacancies due to their drift and line arrangement abilities.

On the other hand, in Ti/CeO_2−x_/ZnO/Pt heterostructure memory devices, a very thin interfacial TiO layer is formed at Ti/CeO_2−x_ interface as obvious from HRTEM image (Fig. 1c) and as suggested by our previous study [37]. Gibbs energy of formation of TiO (− 944 kJ/mol) is relatively larger than that of CeO_2−x_ (− 1024 kJ/mol); hence, although Ti due to its high oxygen affinity captures oxygen ions from CeO_2−x_ to form interfacial TiO layer, a part of oxygen ions returns back to CeO_2−x_ layer in the absence/presence of an external negative field [38]. Gibbs energy of oxide formation for TiO and ZnO are − 944 kJ/mol and − 318.52 kJ/mol respectively. Accordingly, one can obtain Gibbs energy of oxide formation for (1/2)CeO_2_ = − 512 kJ/mol. Comparing with ZnO, oxygen affinity of Ce is little higher than that of Zn so oxygen ions diffuse from ZnO to CeO_2−x_ layer and then to TiO layer from where these ions can migrate to TE, leaving oxygen vacancies in the oxide layers. Consequently, all oxygen ions gather at top interface and conducting filaments with oxygen vacancies are formed between the electrodes. In the presence of opposite biasing polarity, oxygen ions are sent back to the oxide layers, resulting in the filling of oxygen vacancies leading to filament rupture.

The work functions of top Ti and bottom Pt electrodes are 4.33 and 5.65 eV respectively [39]. Electron affinity and work function of ZnO (3.37 eV and 4.35 eV) are higher than those of CeO_2_ (3.50 eV and 3.2 eV) [40]. So an energy barrier at the ZnO/CeO_2−x_ interface is expected, like the Schottky barrier. In the positive voltage regime, electrons cannot be easily injected through the defects in CeO_2_ by Pt bottom electrode to the ZnO layer because work function of ZnO is higher than CeO_2_. That is why electrons are less capable of drifting from ZnO to Ti top electrode, as Ti is unable to attract oxygen ions from ZnO due to their similar work functions. The barrier height at the top Ti/ZnO and CeO_2−x_/Pt bottom interfaces is respectively 0.05 eV and 2.45 eV, barrier height at CeO_2_/Pt bottom interface is higher so electrons cannot be triggered easily from metal to dielectric, which results in the formation of a Schottky barrier at the bottom interface [41].

**Fig. 8:**
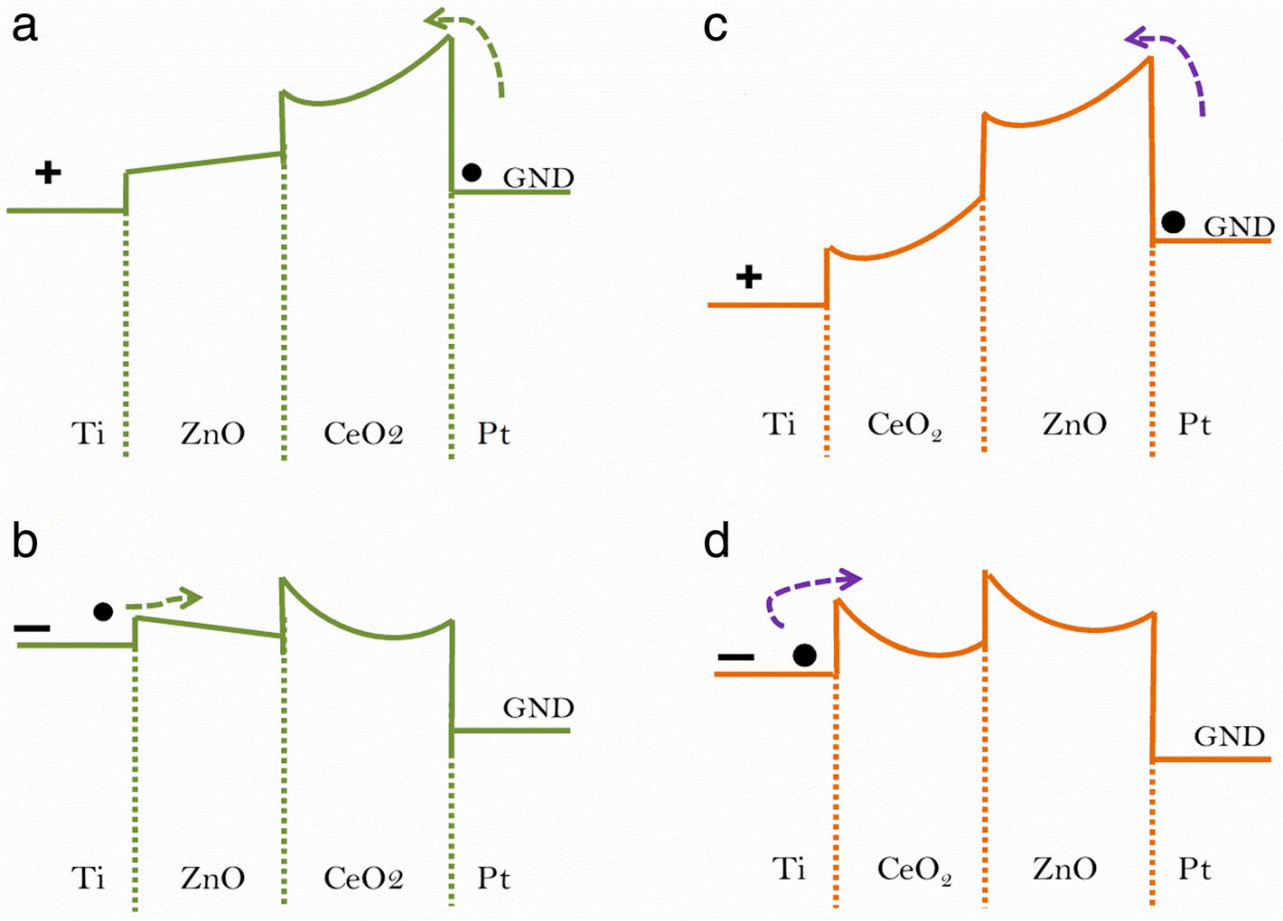
Schematic diagrams for the conduction band offset of **a**, **b** Ti/ZnO/CeO_2−x_/Pt and **c**, **d** Ti/CeO_2−x_/ZnO/Pt heterostructure memory devices. Arrows represent electrons drift direction according to switching polarities

However, the barrier height of top Ti/ZnO interface is negligibly small due to similar work functions, but it is much higher at the bottom CeO_2−x_/Pt interface that is why polarity of biasing field is not sufficient for balancing the barrier heights of the two interfaces; consequently, the endurance and switching characteristics of Ti/ZnO/CeO_2−x_/Pt heterostructure are not so good at positive polarity of applied bias. When negative voltage sweep is applied to Ti top electrode, the electron injection from Ti TE is unable to control the barrier at Ti/ZnO interface because no Schottky barrier is formed at top Ti/ZnO interface in the Ti/ZnO/CeO_2−x_/Pt heterostructure as shown in Fig. 8a, b.

In the positive voltage region, on the other hand, electrons can be easily injected through the defects in ZnO from Pt electrode to the CeO_2−x_ layer. These electrons are then drifted from CeO_2−x_ layer to Ti top electrode. The barrier heights of top Ti/CeO_2−x_ (1.13 eV) and bottom ZnO/Pt (2.28 eV) interfaces suggest a Schottky emission as shown in Fig. 8c, d. When a negative voltage is swept to Ti top electrode, electron injection from top electrode is controlled by this Schottky barrier at Ti/CeO_2−x_ interface, because trapping and de-trapping phenomena can easily occur at the lower barrier (1.13 eV). Oxygen ions can be migrated to Ti/CeO_2−x_ interface by applying a positive voltage. The RS mechanism in Ti/CeO_2−x_/ZnO/Pt heterostructure memory device can be explained by the creation and dissolution of conducting filaments with oxygen vacancies in the oxide layers [41]. It means that oxygen ions can thus move back and forth between Ti/CeO_2−x_ interface and oxide layers by two opposite polarities of the external bias. When a positive voltage is swept on Ti electrode, oxygen ions are drifted from CeO_2−x_/ZnO to Ti/CeO_2−x_ interface. The conducting filaments with oxygen vacancies are formed in the oxide layer, and consequently, resistance state is switched from OFF- (HRS) to ON-state (LRS). When a negative voltage is swept on Ti TE, process of de-trapping is started and oxygen ions gathered at Ti/CeO_2−x_ interface are moved back toward the bottom electrode. The conducting filaments are ruptured due to the migration of oxygen ions. The device is thus switched back again into HRS. Based on the current results, we have investigated the effect of device heterostructure such as CeO_2−x_/ZnO and ZnO/CeO_2−x_ and electroforming polarity on resistive switching parameters for possible applications in resistive random access memory devices. We have noticed that both device structures and their electroforming polarity pose significant influence on switching parameters such as electroforming voltage, memory window, and uniformity in SET/RESET voltages. However, more attention is needed to achieve faster programing/erasing time, higher scalability, electroforming-free, and low cast devices in future research. In particular, work is needed in choosing suitable electrode material, utilizing either nanocrystals or metal ions embedded in an insulating layer and fabricating device on buffer layer structures.

## Conclusions

In conclusion, deep investigations on the RS behavior have been made by changing the morphology of bilayer ZnO/CeO_2−x_ and CeO_2−x_/ZnO heterostructures and sign of electroforming polarities. Significant impact is noticed on the performance, endurance characteristics, electroforming voltages, and uniformity in the operational voltages. Experimental results reveal the formation of TiO interfacial layer in Ti/CeO_2−x_/ZnO/Pt heterostructure on applying bias of positive polarity, and CeO_2−x_ layer during negative polarity serves as an oxygen reservoir in Ti/ ZnO/CeO_2−x_/Pt heterostructures. Collectively, it can play an important role for the improvement of uniformity and repeatability of RS parameters. Dominant conduction mechanism in HRS was electrode-limited Schottky emission at a high field region. Temperature dependence of LRS and HRS resistances lead to the conclusion that observed RS mechanism is based on the movement of oxygen vacancies under the applied voltage.

## Abbreviations

BRS: Bipolar resistive switching
DC: Direct current
HRS: High resistance state
HRTEM: High-resolution transmission electron microscopy
LRS: Low resistance state
MOM: Metal-oxide-metal
RRAM: Resistive random access memory
RS: Resistive switching
TE: Top electrode
URS: Unipolar resistive switching
